# Functional proteomic and phosphoproteomic profiling of routine FFPE CNS tumor tissue complements genomic and epigenomic characterization

**DOI:** 10.1007/s00401-026-03091-6

**Published:** 2026-09-25

**Authors:** Dennis Friedel, Rhaissa Ribeiro da Silva, Ivan Abdulrazak Ahmed, Beatriz Martins Wolff, Anna Neuerburg, Ramona Irsevic, Shiva Ahmadi, Ashok Kumar Jayavelu, Andreas E. Kulozik, Olaf Witt, Stefan M. Pfister, Sandro M. Krieg, Wolfgang Wick, Andreas von Deimling, David E. Reuss, Felix Sahm, Philipp Sievers

**Affiliations:** 1 Department of Neuropathology, Institute of Pathology, University Hospital Heidelberg, Heidelberg, Germany; 2 Clinical Cooperation Unit Neuropathology, German Consortium for Translational Cancer Research (DKTK), German Cancer Research Center (DKFZ), Heidelberg, Germany; 3 Proteomics Core Facility, German Cancer Research Center (DKFZ), Heidelberg, Germany; 4 Hopp Children’s Cancer Center Heidelberg (KiTZ), Heidelberg, Germany; 5 Department of Pediatric Oncology, Hematology, Immunology and Pulmonology, University Hospital Heidelberg, Heidelberg, Germany; 6 Clinical Cooperation Unit Pediatric Leukemia, German Consortium for Translational Cancer Research (DKTK), German Cancer Research Center (DKFZ), Heidelberg, Germany; 7 Clinical Cooperation Unit Pediatric Oncology, German Consortium for Translational Cancer Research (DKTK), German Cancer Research Center (DKFZ), Heidelberg, Germany; 8 National Center for Tumor Diseases (NCT), NCT Heidelberg, a partnership between DKFZ and Heidelberg University Hospital, Heidelberg, Germany; 9 Division of Pediatric Neurooncology, German Consortium for Translational Cancer Research (DKTK), German Cancer Research Center (DKFZ), Heidelberg, Germany; 10 Department of Neurosurgery, Heidelberg University Hospital, Heidelberg, Germany; 11 Clinical Cooperation Unit Neurooncology, German Consortium for Translational Cancer Research (DKTK), German Cancer Research Center (DKFZ), Heidelberg, Germany; 12 Department of Neurology and Neurooncology Program, European Center for Neurooncology (EZN), Heidelberg University, University Hospital Heidelberg, Heidelberg, Germany

**Keywords:** Phosphoproteomics, FFPE, Glioblastoma, Molecular diagnostics, *EGFR* amplification

## Abstract

Genomic and DNA methylation-based analyses have transformed the classification of central nervous system (CNS) tumors by enabling robust molecular diagnosis and subclassification. However, these approaches primarily define tumor identity and do not directly capture the functional state of intracellular signaling networks. Proteomic and phosphoproteomic profiling provide complementary molecular layers by measuring protein abundance and site-specific phosphorylation. Recent methodological advances have made a comprehensive analysis of formalin-fixed, paraffin-embedded (FFPE) tissue increasingly feasible, raising the question of how functional proteomic information can complement established molecular diagnostics. Here, we applied an FFPE-compatible workflow for proteomic and phosphoproteomic profiling of ten comprehensively characterized glioblastomas, including five *EGFR*-amplified and five non-amplified tumors. Mass spectrometry generated robust proteomic and phosphoproteomic coverage across all cases. Global proteomic profiling revealed group-associated protein abundance and pathway differences, including increased EGFR abundance in *EGFR*-amplified tumors, while showing substantial intertumoral overlap. Protein-abundance-adjusted phosphoproteomic profiles similarly showed partial group-level separation with substantial intertumoral overlap. Differential analysis of protein-abundance-adjusted phosphoproteomic data identified 443 phosphosites based on predefined exploratory statistical criteria. Kinase-substrate enrichment further revealed coordinated EGFR- and SRC-family-associated signaling with marked variability across individual tumors. These findings demonstrate that routine FFPE tissue retains biologically coherent functional information at both the protein abundance and phosphosignaling levels that can be interpreted alongside genomic and epigenomic data. Phosphoproteomics therefore represents an orthogonal functional layer that may complement established molecular characterization of CNS tumors.

**Supplementary Information:**

The online version contains supplementary material available at https://doi.org/10.1007/s00401-026-03091-6.

## Introduction

The classification of central nervous system (CNS) tumors has been transformed by the integration of molecular diagnostics with conventional histopathology [11]. Genomic sequencing, copy number analysis, and genome-wide DNA methylation profiling have substantially improved diagnostic accuracy, refined tumor taxonomy, and enabled the recognition of biologically distinct tumor types that cannot be reliably resolved by morphology alone [4, 11, 14]. Consequently, molecular diagnostics have become central to defining tumor identity, lineage, recurrent driver alterations, and genomic architecture.

Despite these advances, established molecular assays primarily characterize relatively stable layers of tumor biology. Although genomic and epigenomic analyses define molecular identity with high precision, they do not directly capture the functional state of intracellular signaling pathways. This may contribute to the limited impact that these refined diagnoses have had so far in the clinical setting. Information on functional consequences may help address this gap. Protein abundance and site-specific phosphorylation provide complementary information by reflecting the integrated consequences of genomic alterations, post-transcriptional regulation, and cellular signaling. Proteomic and phosphoproteomic profiling therefore offer the opportunity to connect molecular classification with pathway activity [13, 23].

The widespread use of formalin-fixed, paraffin-embedded (FFPE) tissue has historically represented a major obstacle to proteomic analysis in routine pathology. However, methodological advances in tissue processing, phosphopeptide enrichment, and mass spectrometry have established that comprehensive proteomic and phosphoproteomic information can be recovered from FFPE specimens [7, 22, 26]. The remaining challenge is therefore not solely technical feasibility, but how such functional molecular information can be integrated with established diagnostic frameworks and interpreted in the context of genomically and epigenomically defined tumors.

Glioblastoma provides a suitable model to evaluate this concept because its diagnosis routinely integrates histopathology with genomic, copy number, and DNA methylation-based analyses, while *EGFR amplification* represents one of its most frequent and biologically well-characterized genomic alterations [11, 23]. Rather than aiming to identify novel biomarkers or molecular subclasses from a small cohort, this proof-of-concept study asked whether proteomic and phosphoproteomic profiling of routine FFPE tissue can provide biologically coherent functional information alongside established molecular diagnostics, whether known signaling consequences of *EGFR amplification* can be recovered, and whether phosphoproteomics captures functional heterogeneity beyond binary genomic classification. Although demonstrated here in glioblastoma, this framework may be applicable to the molecular characterization of CNS tumors more broadly.

## Materials and methods

### Patient samples

Archived FFPE tumor material from ten patients with glioblastoma, IDH-wildtype, was retrospectively selected from the Department of Neuropathology, University Hospital Heidelberg, Germany. All cases were reviewed histologically and had undergone integrated molecular characterization as part of the diagnostic workflow. The cohort comprised five tumors with *EGFR amplification* and five tumors without *EGFR amplification;* clinical and molecular characteristics are summarized in Supplementary Table 1. Selection was based on the availability of sufficient FFPE material, adequate tumor content, and complete molecular data, including genome-wide DNA methylation profiling and targeted sequencing. This study was approved by the ethics committee of Heidelberg University (S-318/2022). Clinical and molecular data were pseudonymized before analysis.

### Nucleic acid extraction

Genomic DNA was extracted from FFPE tissue sections enriched for tumor cell content based on neuropathological review. DNA extraction was performed using the automated Maxwell RSC platform (Promega, Madison, WI, USA) with the Maxwell RSC FFPE Plus DNA Kit according to the manufacturer's instructions. DNA concentration was quantified using the Qubit dsDNA Broad Range Assay Kit (Thermo Fisher Scientific, Waltham, MA, USA). Extracted DNA was subsequently used for genome-wide DNA methylation profiling and targeted next-generation sequencing.

### DNA methylation profiling and copy number analysis

Genome-wide DNA methylation profiling was performed using Illumina Infinium MethylationEPIC BeadChip array according to established protocols, as previously described [4]. Raw intensity data (IDAT files) were processed using computational pipelines implemented in R. Data preprocessing included background correction, dye-bias correction, normalization, and quality control assessment [4]. Technical variation associated with array type was corrected using linear modeling approaches. DNA methylation-based tumor classification was performed using established reference datasets and classification algorithms [18] (Heidelberg Brain Tumor Classifier, version 12.8). The assigned methylation class and the corresponding calibrated classifier score for each individual tumor are provided in Supplementary Table 1. Copy number alteration profiles were derived from methylation array data using the conumee package in R. Focal genomic alterations were assessed by manual inspection of log2 ratio plots and integrated with sequencing-based findings.

### Targeted DNA sequencing

Targeted next-generation sequencing was performed using a custom-designed enrichment-based panel covering coding exons and selected intronic regions of 220 genes associated with central nervous system tumors [17]. Sequencing libraries were generated according to the manufacturer's protocols and sequenced on Illumina NovaSeq 6000 platforms. Sequence reads were aligned to the human reference genome GRCh38 (hg38) using the Burrows–Wheeler Aligner (BWA). Variant calling included single-nucleotide variants, small insertions/deletions, and selected structural alterations including gene fusions. Variants with an allele frequency below 5% were excluded from further analysis. Variant interpretation and pathogenicity assessment were performed according to American College of Medical Genetics and Genomics (ACMG) guidelines.

### FFPE tissue processing and protein extraction

FFPE tissue processing and protein extraction were performed using an optimized plate-based workflow as described below. Tissue punches were transferred to randomized positions in an Eppendorf twin.tec^®^ PCR Plate 96 LoBind^®^ (Eppendorf, Hamburg, Germany; cat. no. 0030129512). Each punch was incubated in 100 µL of 4% (w/v) sodium dodecyl sulfate (SDS), and the plate was sealed using domed strip caps. Small cylindrical Gyuto Beads 2 × 2 (PreOmics, art. no. P.0.00154) were placed in each sample-containing well and in the directly adjacent wells above, below, and on either side. The plate was processed using a BeatBox^®^ tissue homogenizer (PreOmics, Germany) set to high force for 10 min. In the instrument, an alternating magnetic field drives the magnetic disruption elements to generate mechanical impact and shear forces, facilitating disruption of the tissue and surrounding paraffin. No conventional xylene-based deparaffinization was performed. The samples were subjected to four alternating cycles of mechanical tissue disruption and heat treatment. Each cycle consisted of 10 min of BeatBox processing followed by incubation for 20 min at 95 °C in a thermocycler with the heated lid set to 95 °C. Between the mechanical disruption and heating steps, the plate was centrifuged for 60 s at 800 rpm to collect lysate and tissue material at the bottom of the wells. This alternating procedure was repeated for a total of four cycles. Following the fourth cycle, the plate was centrifuged once more, and the protein-containing lysates in 4% SDS were transferred to a new 96-well plate.

### Protein quantification and normalization

An aliquot of each lysate was removed for protein concentration determination using the Pierce™ BCA Protein Assay Kit (Thermo Fisher Scientific). Based on the measured protein concentration, an appropriate volume of lysate was transferred into a Protein LoBind microcentrifuge tube. The transferred amount was selected to achieve a target protein concentration of 1.5 g/L after resuspension in 50 µL RIPA buffer. The selected lysate volume was dried to completeness in a vacuum concentrator at 45 °C. Dried samples were resuspended in 50 µL RIPA buffer and sonicated using a Bioruptor instrument (Diagenode) for 5 min with alternating 30 s ON and 30 s OFF cycles.

### Automated SP3-based protein digestion

Following sonication, the samples were transferred to a clear, skirted PCR 96 SuperPlate (Kisker Biotech, AB-2800; art. no. 017219) and processed using an automated single-pot, solid-phase-enhanced sample preparation (SP3) workflow on an Agilent Bravo liquid-handling platform. A protein input of 55 µg per sample was used for the SP3 workflow. A 1:1 mixture of Sera-Mag SpeedBead Magnetic Carboxylate A and B particles (Cytiva) was used. Before use, the beads were removed from the storage solution and washed three times with LC–MS-grade water to remove the residual storage buffer. Proteins were reduced with tris(2-carboxyethyl)phosphine (TCEP) and alkylated with 2-chloroacetamide (CAA) to final concentrations of 10 mM and 40 mM, respectively. Protein binding to the magnetic beads was induced by addition of 100% ethanol to a final concentration of 50%. The bead-bound proteins were subsequently washed twice with 80% ethanol and once with 100% acetonitrile, followed by resuspension in 100 mM triethylammonium bicarbonate (TEAB). Proteins were digested on-bead with sequencing-grade modified trypsin (Promega, V5111) at an enzyme-to-protein ratio of 1:10 for 16 h at 37 °C with agitation at 500 rpm on an Eppendorf benchtop ThermoMixer. Following digestion, the plate was placed on a magnetic rack and the peptide-containing supernatant was recovered. An aliquot corresponding to 3 µg of peptides was transferred to an Eppendorf twin.tec^®^ PCR Plate 96 LoBind® (Eppendorf, Hamburg, Germany; cat. no. 0030129512) for global proteome analysis, while the remaining peptide material was retained for phosphopeptide enrichment. Both fractions were dried to completeness in a SpeedVac at 45 °C. The digestion was stopped by vacuum drying without prior acidification.

### Automated phosphopeptide enrichment

Dried peptide samples intended for phosphoproteomic analysis were reconstituted in 20 µL of 0.1% trifluoroacetic acid (TFA) in water, followed by addition of 80 µL of 0.1% TFA in acetonitrile. Samples were vortexed until visible particulates were dissolved, centrifuged at 6,000 × g for 5 min, and transferred to a 96-well PCR plate. Phosphopeptides were enriched using Fe^3^⁺-NTA cartridges on an Agilent AssayMAP Bravo platform with the Phosphopeptide Enrichment v2.1 workflow. The cartridges were primed with 0.1% TFA in 50% acetonitrile and equilibrated with 0.1% TFA in 80% acetonitrile. Following sample loading and washing, bound phosphopeptides were eluted using 1% ammonium hydroxide in water at approximately pH 11. The phosphopeptide eluate was used for LC–MS/MS analysis, while the corresponding flow-through was retained. Both fractions were dried to completeness by vacuum centrifugation using a SpeedVac at 45 °C and stored at −20 °C. Before LC–MS/MS analysis, the dried phosphopeptides were resuspended in 17 µL of water containing 50 mM citric acid and 0.1% TFA. Global-proteome samples were reconstituted in 36 µL sample buffer containing 97.4% water, 2.5% hexafluoro-2-propanol (HFIP), and 0.1% TFA.

### Quality control

An MCF7 reference sample was processed alongside the tumor samples through the complete SP3 digestion and Fe^3^⁺-NTA phosphopeptide-enrichment workflow and served as a process control for sample preparation and phosphopeptide enrichment. A HeLa peptide standard (20 ng) was injected at regular intervals, approximately every 14 LC–MS injections, to monitor instrument performance and measurement stability. Each biological tumor sample was analyzed once per acquisition workflow without technical replicate injections.

### LC–MS/MS analysis

Global-proteome and phosphopeptide-enriched samples were analyzed using an UltiMate 3000 UHPLC system directly coupled to an Orbitrap Exploris 480 mass spectrometer (Thermo Fisher Scientific). Peptides were separated on a nanoEase M/Z Peptide BEH C18 analytical column (130 Å pore size, 1.7 µm particle size, 75 µm × 250 mm; Waters, 186,008,795) maintained at 35 °C. For phosphoproteomic analysis, peptides were online-desalted on an Acclaim PepMap300 C18 trapping cartridge (5 µm, 300 Å wide pore; Thermo Fisher Scientific) for 3 min at a flow rate of 30 µL/min using 0.05% TFA in water. Analytical separation was performed at 300 nL/min using solvent A (0.1% formic acid in water) and solvent B (0.1% formic acid in acetonitrile). For global proteome analysis, 1 µg of peptide digest was injected and analyzed using a 90 min data-independent acquisition (DIA) method. The LC gradient was run at 300 nL/min and 35 °C. Starting at 2% solvent B, the composition was held at 2% B until 3 min, increased to 4% B at 4 min, and then linearly increased to 30% B by 76 min. Solvent B was increased to 80% at 77 min and held at 80% until 79 min, followed by a decrease to 2% B at 80 min and re-equilibration until 90 min. MS1 spectra were acquired in positive-ion mode at an Orbitrap resolution of 120,000 over an m/z range of 350–1,400, using a normalized AGC target of 300% (absolute AGC value 3 × 10⁶) and a maximum injection time of 45 ms. DIA MS2 acquisition covered the precursor range of approximately 380–880 m/z using 34 variable isolation windows. Precursors were fragmented using higher-energy collisional dissociation (HCD) with a normalized collision energy of 28%. MS2 spectra were recorded at an Orbitrap resolution of 30,000 with a normalized AGC target of 1,000% (absolute AGC value 1 × 10⁶) and automatic maximum injection time. The calculated DIA cycle time was approximately 2.3–2.5 s. For phosphoproteomic analysis, 15 µL, corresponding to approximately 90% of the phosphopeptide-enriched sample, was injected and analyzed using a 90 min data-dependent acquisition (DDA) method. Each sample was followed by a 22 min wash run to minimize carry-over between samples. The analytical gradient was run at 300 nL/min and 35 °C using solvent A (0.1% formic acid in water) and solvent B (0.1% formic acid in acetonitrile). Starting at 2% B, the composition was held at 2% until 3 min and then linearly increased to 28% B by 76 min. Solvent B was increased to 78% at 77 min and held at 78% until 79 min, followed by a decrease to 2% B at 80 min and re-equilibration until 90 min. MS1 spectra were acquired in positive-ion mode at an Orbitrap resolution of 60,000 over an m/z range of 380–1,400, with a normalized AGC target of 200% (absolute AGC value 2 × 10⁶) and automatic maximum injection time. Data-dependent acquisition was performed using a fixed cycle time of 1.5 s between master scans. Precursor ions with charge states 2–6 were selected for fragmentation; unassigned and singly charged features were excluded. An intensity threshold of 6 × 10^4^ was applied. Precursors were isolated using a 1.0 m/z window and fragmented by HCD at a normalized collision energy of 30%. MS2 spectra were acquired at an Orbitrap resolution of 30,000 with a normalized AGC target of 200% (absolute AGC value 2 × 10^5^) and a maximum injection time of 110 ms. Dynamic exclusion was set to 25 s with a ±10 ppm mass tolerance, and isotope exclusion was enabled.

### Processing of global proteome and phosphoproteomic data

Raw spectra from global proteomic DIA and phosphoproteomic DDA acquisitions were processed on a Linux system with 128 physical cores (AMD EPYC 7503 32-Core Processor) and 256 GB of RAM.

The command-line version of MaxQuant (v.2.4.2.0) [19] was used to process phosphoproteomic DDA data. Peptide-spectrum matches (PSMs) and proteins were filtered at a false discovery rate (FDR) of 1%. Label-free quantification was performed with a minimum ratio count of 1. Variable modifications included oxidation (M), protein N-terminal acetylation, deamidation (NQ), and phosphorylation (STY). Match between runs (MBR) was enabled to increase quantification coverage across samples based on retention-time alignment and accurate mass.

Global proteomic DIA data were processed using the command-line version of DIA-NN v2.3.1 with default settings and in library-free mode [5]. A minimum peptide length of 7 and a maximum peptide length of 30 amino acids were used. Fragment ion m/z values were restricted to 200–1,800, and precursor m/z values to 300–1,800. Only proteotypic peptides were considered for protein quantification, and a *q*-value threshold of 0.05 was applied at the precursor and protein levels. The second-pass search (“--reanalyze”) option was enabled. In addition, DIA-NN normalization using QuantUMS was enabled to obtain globally normalized protein intensities [6].

For both processing workflows, raw spectra were searched against canonical human protein sequences obtained from the UniProt database on January 16, 2025 [21], comprising 20,547 proteins and 20,310 genes. Trypsin was specified as the digestion enzyme, allowing up to two missed cleavages.

### Preprocessing and quality control of global proteomic and phosphoproteomic data

Further analyses were performed in R version 4.5.2. DIA-NN-normalized global proteomic intensities and phosphoproteomic intensities were log₂-transformed.

For global proteomic data, protein intensities mapping to unique genes and meeting a *q*-value threshold of < 0.01 were retained for further analysis, and samples with fewer than 5000 identified proteins were excluded. For phosphoproteomic data, phosphosites marked as contaminants or reverse hits, as well as phosphosites with a localization probability below 75%, were excluded. Samples with fewer than 3500 identified phosphosites were excluded from further analysis.

For both datasets, missing values were evaluated separately within *EGFR*-amplified and non-amplified tumors. Proteins or phosphosites identified in fewer than 75% of samples in both biological groups were excluded from further analysis. Phosphoproteomic intensities were additionally normalized by median centering across samples. To account for differences in total protein abundance, phosphosite intensities were subsequently adjusted by subtracting the log₂-transformed abundance of the corresponding protein measured in the matched global proteome. Remaining missing values were imputed using the left-censored MinProb method implemented in the R package imputeLCMD.

### Downstream analysis of global proteome and phosphoproteomic data

Normalized and imputed log₂ protein intensities and protein-abundance-adjusted and imputed phosphosite intensities were used for downstream analyses including dimensional reduction by principal component analysis (PCA), differential abundance analysis, gene set enrichment analysis (GSEA), and kinase activity inference.

To evaluate whether *EGFR* amplification status contributed to data variance, a one-way ANOVA was performed on PC scores 1–10 to test for significant associations and estimate the proportion of variance (poV) explained.

Differential abundance of proteins and phosphosites between *EGFR*-amplified and non-amplified tumors was assessed using the R-package *limma* [16]. Proteins/phosphosites of interest were defined by an absolute log₂ fold change > 0.58 and a nominal P value < 0.05.

GSEA was conducted on the global proteome to further evaluate biological differences between the compared groups. Therefore, t-statistics obtained from differential abundance analysis were analyzed by fast gene set enrichment analysis (fgsea; https://doi.org/10.18129/B9.bioc.fgsea) using 10,000 permutations, with a minimum gene set size of 5 and a maximum of 300. The input was tested against Hallmark gene sets obtained from MSigDB. Hallmarks of interest were selected based on an adjusted *P* value < 0.05.

Kinase activity inference was conducted on phosphoproteomic data using the functions provided by the R package decoupler [3]. The latest Kinase–Substrate (KS) databases (13/08/2025), including KS relationships from ProtMapper, were downloaded from the OmniPath archive (https://archive.omnipathdb.org). Subsequently, ‘Virtual Inference of Protein activity by Enriched Regulon analysis’ (VIPER) [2] was used to calculate kinase activities. The t-statistics from the differential abundance analysis were used as input to compare kinase activities between the groups. Kinases were filtered for a P value < 0.05, and the 15 highest- and 15 lowest-scoring kinases were selected for investigation at the single-sample level. Here, log₂-normalized, protein-abundance-adjusted, and imputed phosphosite intensities were used for kinase activity inference at the single-sample level. For both approaches, kinase activity scores were estimated only for kinases with a minimum of 5 substrates.

To infer activity in therapeutically relevant pathways at the single-sample level, kinases with a *P* value < 0.05 were subjected to overrepresentation analysis (ORA) using the functions provided by the R package clusterProfiler [25] with a minimum gene set size of 5 and a maximum of 300, with Benjamini–Hochberg correction for *P* values. In total, 177 Reactome gene sets were curated for ORA and assigned to predefined therapeutically relevant signaling categories [15], including EGFR/ERBB signaling, ATR−CHK−WEE1/DNA damage signaling, PI3K−AKT−mTOR signaling, RAS−RAF−MEK−ERK/MAPK signaling, VEGF/angiogenic signaling, CDK/cell cycle signaling, SRC-family signaling, and PKC signaling (Supplementary Table 2). Kinases were assigned to the corresponding signaling categories as summarized in Supplementary Table 2. Subsequently, pathways of interest were selected based on an adjusted *P* value < 0.05.

Given the exploratory nature and limited sample size of the study, pathway- and kinase-level analyses were considered hypothesis-generating.

For illustration of a potential case-level reporting framework, phosphoproteomic findings from one representative tumor were used. The prototype report integrated analytical quality-control metrics, Reactome pathway enrichment, and representative phosphosites assigned to signaling categories. All components of the report were derived from the individual sample without requiring comparison with the remaining cohort. This analysis was considered exploratory and was not intended to establish diagnostic thresholds or predict therapeutic response.

## Results

### Routine FFPE glioblastoma tissue yields robust proteomic and phosphoproteomic profiles

**Fig. 1:**
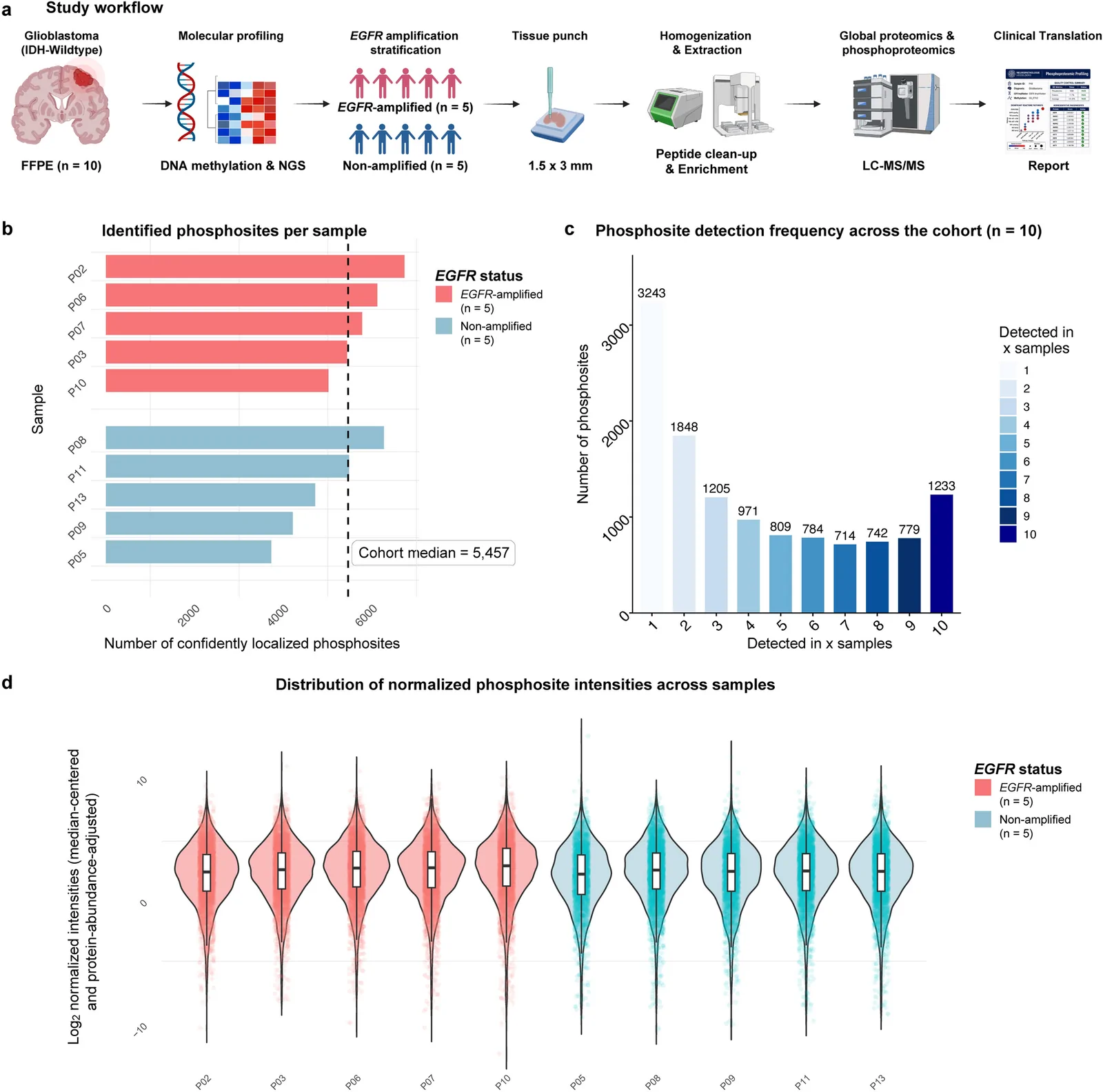
Study workflow and quality assessment of FFPE phosphoproteomic profiling. **a** Schematic overview of the study design. Ten molecularly characterized glioblastomas are stratified according to *EGFR* amplification status into five *EGFR*-amplified and five non-amplified tumors and are subjected to global proteomic and phosphoproteomic profiling (created in BioRender. Ribeiro da Silva, R. (2026)). **b** The bar plot shows the number of confidently localized phosphosites identified per sample. The dashed line indicates the cohort median. **c** Phosphosite detection frequency across the cohort shows the number of phosphosites detected in one to ten individual tumors. **d** Violin plots show the distribution of log₂-transformed, median-centered and protein-abundance-adjusted phosphosite intensities across individual samples. Comparable intensity distributions indicate consistent quantitative performance across the cohort

We first assessed the analytical performance of proteomic and phosphoproteomic profiling in diagnostically archived FFPE tissue. Ten glioblastomas with complete histological, DNA methylation, copy number, and targeted sequencing data were included, comprising five *EGFR*-amplified and five non-amplified tumors (Fig. 1a). Quantitative global proteomic and phosphoproteomic data were obtained from all cases.

Global proteomic profiling yielded broad and reproducible protein coverage, with a median of 7647 proteins identified per tumor and 5417 proteins detected in all ten samples (Supplementary Figure 1). Normalized protein intensity distributions were comparable across individual tumors, supporting consistent quantitative performance of the global proteomic dataset.

Phosphoproteomic analysis identified a total of 13,467 phosphosites following MaxQuant processing, with a cohort median of 5457 confidently localized phosphosites per sample and a range of 3724–6729 (Fig. 1b). Phosphosite detection-frequency analysis showed that a substantial fraction of sites was reproducibly detected across multiple tumors, including 1233 phosphosites identified in all ten samples (Fig. 1c). Following median centering and adjustment for corresponding protein abundance, phosphosite intensity distributions were comparable across individual tumors (Fig. 1d). Together, these quality-control metrics demonstrate robust and quantitatively comparable proteomic and phosphoproteomic coverage across the cohort.

### Global proteomic profiling reveals group-associated protein abundance and pathway differences

**Fig. 2:**
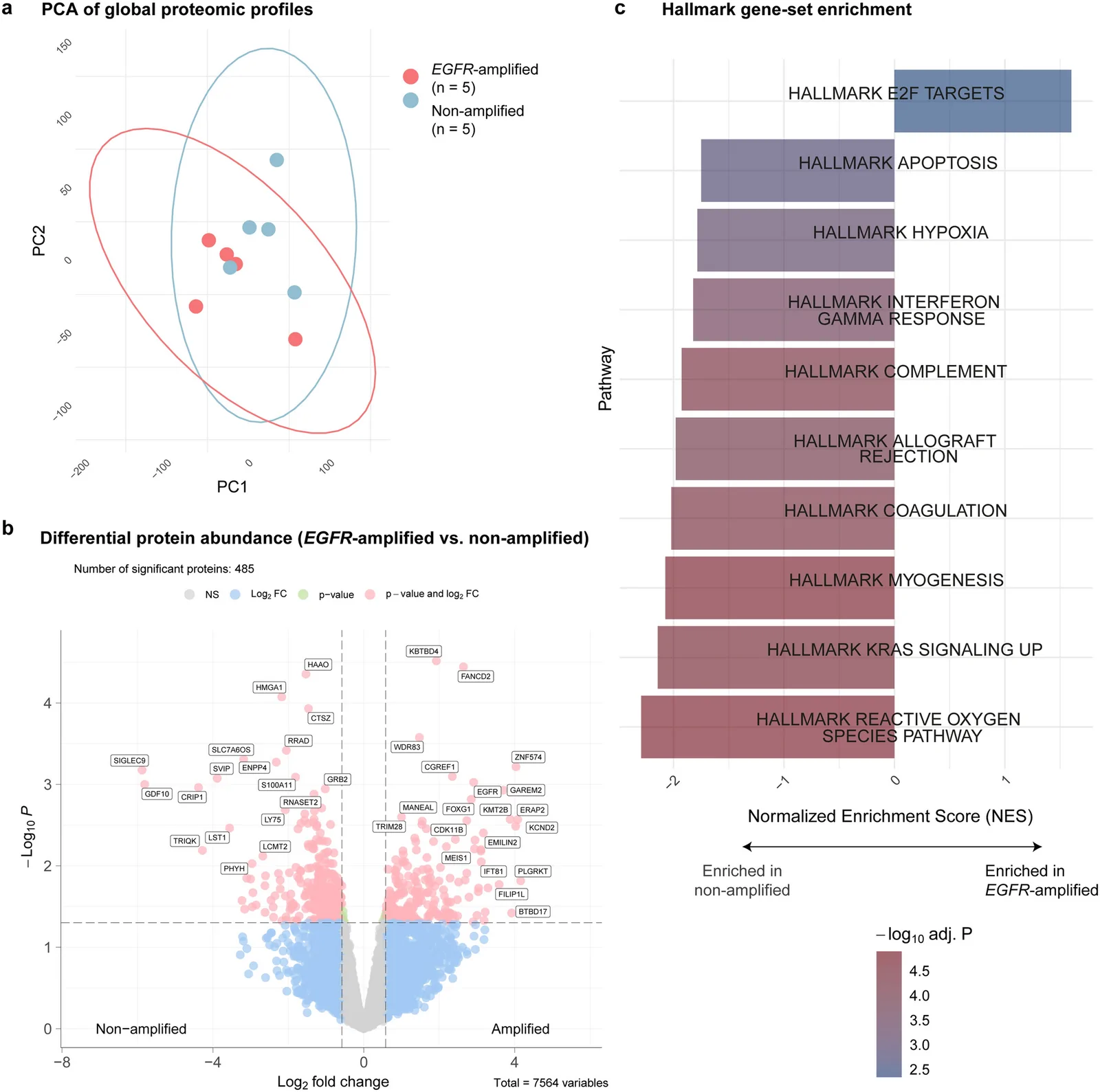
Global proteomic profiling reveals group-associated protein abundance and pathway differences. **a** Principal component analysis (PCA) of global proteomic profiles from *EGFR*-amplified (*n = *5) and non-amplified (*n = *5) glioblastomas. Samples show substantial overlap between groups, indicating considerable intertumoral proteomic heterogeneity. **b** The volcano plot shows differential protein abundance between *EGFR*-amplified and non-amplified tumors. The x-axis represents the log₂ fold change and the y-axis the −log₁₀ nominal P value. Positive log₂ fold changes indicate enrichment in *EGFR*-amplified tumors, whereas negative values indicate enrichment in non-amplified tumors. A total of 485 proteins meet the predefined exploratory criteria of an absolute log₂ fold change > 0.58 and nominal *P < *0.05. **c** Hallmark gene-set enrichment analysis of global protein abundance differences between the two groups. Bars represent normalized enrichment scores (NES), with positive values indicating enrichment in *EGFR*-amplified tumors and negative values indicating enrichment in non-amplified tumors. Color indicates the −log₁₀ Benjamini–Hochberg-adjusted P value

We next examined whether global protein abundance patterns were associated with *EGFR-amplification* status. Unsupervised principal component analysis showed substantial overlap between *EGFR*-amplified and non-amplified tumors, indicating considerable intertumoral heterogeneity at the global protein level (Fig. 2a). Differential protein abundance analysis identified 485 proteins meeting predefined exploratory statistical criteria among 7564 tested proteins, with EGFR among the proteins enriched in *EGFR*-amplified tumors (Fig. 2b; Supplementary Table 3). Hallmark gene-set enrichment analysis further revealed group-associated differences in biological programs. E2F target proteins were enriched in *EGFR*-amplified tumors, whereas several stress-, immune-, and microenvironment-associated programs, including hypoxia, interferon-γ response, complement, reactive oxygen species signaling, and apoptosis, were enriched in non-amplified tumors (Fig. 2c). Together, these findings indicate that *EGFR* amplification status is associated with broader proteomic differences while substantial heterogeneity remains within both molecular groups.

### Protein-abundance-adjusted phosphoproteomic profiles show partial separation according to *EGFR* amplification status

**Fig. 3:**
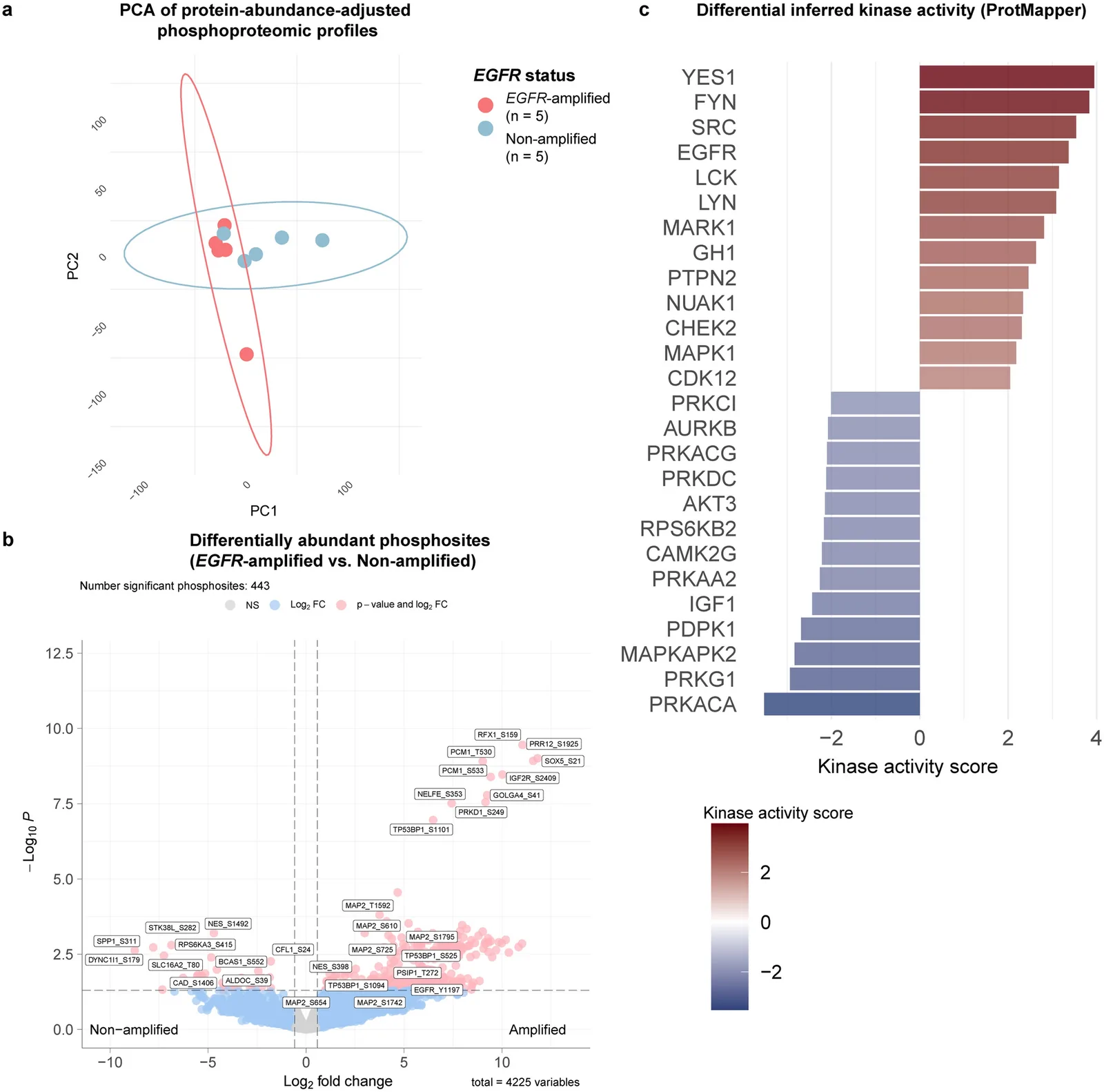
Protein-abundance-adjusted phosphoproteomic alterations associated with *EGFR* amplification reveal distinct but heterogeneous signaling states. **a** Principal component analysis of protein-abundance-adjusted phosphoproteomic profiles from *EGFR*-amplified (*n = *5) and non-amplified (*n = *5) glioblastomas. Samples show substantial overlap between groups with partial group-associated separation. **b** Volcano plot showing differential phosphosite abundance between *EGFR*-amplified and non-amplified tumors after adjustment for corresponding protein abundance. The x-axis shows the log₂ fold change and the y-axis the −log₁₀ nominal P value. Positive log₂ fold changes indicate enrichment in *EGFR*-amplified tumors, whereas negative values indicate enrichment in non-amplified tumors. A total of 443 phosphosites meet the predefined exploratory criteria of an absolute log₂ fold change > 0.58 and nominal *P < *0.05. **c** Differential inferred kinase activity based on curated kinase–substrate relationships from ProtMapper using protein-abundance-adjusted phosphoproteomic data. Positive kinase activity scores indicate enrichment in *EGFR*-amplified tumors, whereas negative scores indicate enrichment in non-amplified tumors

We next asked whether site-specific phosphorylation provided an additional functional layer beyond global protein abundance. Unsupervised principal component analysis was used to examine the dominant sources of variation in the protein-abundance-adjusted phosphoproteomic dataset. The two molecular groups showed substantial overlap, although partial separation remained evident (Fig. 3a). PC1 accounted for 29.9% of the total variance in the protein-abundance-adjusted phosphoproteomic dataset, and *EGFR* amplification status was significantly associated with PC1 scores (*P < *0.05). Thus, *EGFR* amplification status is associated with variation in the protein-abundance-adjusted phosphoproteomic profiles but does not define a uniformly distinct phosphoproteomic state. The remaining overlap and within-group dispersion are consistent with substantial intertumoral heterogeneity and motivated subsequent analyses of individual phosphosites and signaling pathways.

### Phosphoproteomic analysis recovers signaling changes associated with *EGFR* amplification

We next asked whether protein-abundance-adjusted phosphoproteomic profiles captured biologically meaningful signaling changes associated with a well-established genomic alteration. Differential analysis between *EGFR*-amplified and non-amplified tumors identified 443 phosphosites meeting the predefined exploratory criteria of an absolute log₂ fold change > 0.58 and nominal *P < *0.05 (Fig. 3b; Supplementary Table 4). Among phosphosites enriched in *EGFR*-amplified tumors were several sites on TP53BP1, including S1101, together with multiple sites on MAP2 and other proteins. Importantly, EGFR Y1110 and Y1197 remained enriched in *EGFR*-amplified tumors after adjustment for corresponding protein abundance.

Conversely, several phosphosites, including NES S1492, were enriched in non-amplified tumors. These findings indicate that protein-abundance-adjusted phosphoproteomic profiling captures biologically coherent phosphorylation patterns associated with *EGFR amplification.* Given the limited cohort size and multiple-testing burden, individual phosphosites should be considered hypothesis-generating rather than candidate biomarkers.

### Kinase-substrate enrichment reveals EGFR- and SRC-family-associated signaling

To extend the analysis beyond individual phosphosites, we inferred differential kinase activity using kinase-substrate enrichment based on curated ProtMapper annotations (Fig. 3c; Supplementary Table 5). Analysis of the protein-abundance-adjusted phosphoproteomic data identified higher inferred activity of several kinases in *EGFR*-amplified tumors. YES1, FYN, SRC, and EGFR were among the strongest positively enriched kinase signals, together with LCK and LYN, indicating coordinated enrichment of EGFR- and SRC-family-associated signaling.

Conversely, several kinases, including PRKACA, PRKG1, MAPKAPK2, and PDPK1, showed lower inferred activity in *EGFR*-amplified tumors relative to non-amplified tumors. Because kinase-substrate enrichment infers kinase activity from the coordinated behavior of annotated substrates rather than directly measuring enzymatic activity, these findings should be interpreted as pathway-level inferences. Nevertheless, the enrichment of EGFR- and SRC-family-associated kinase signals after adjustment for corresponding protein abundance supports functional signaling differences associated with *EGFR* amplification.

### Case-level phosphoproteomic profiling illustrates a potential reporting framework

**Fig. 4:**
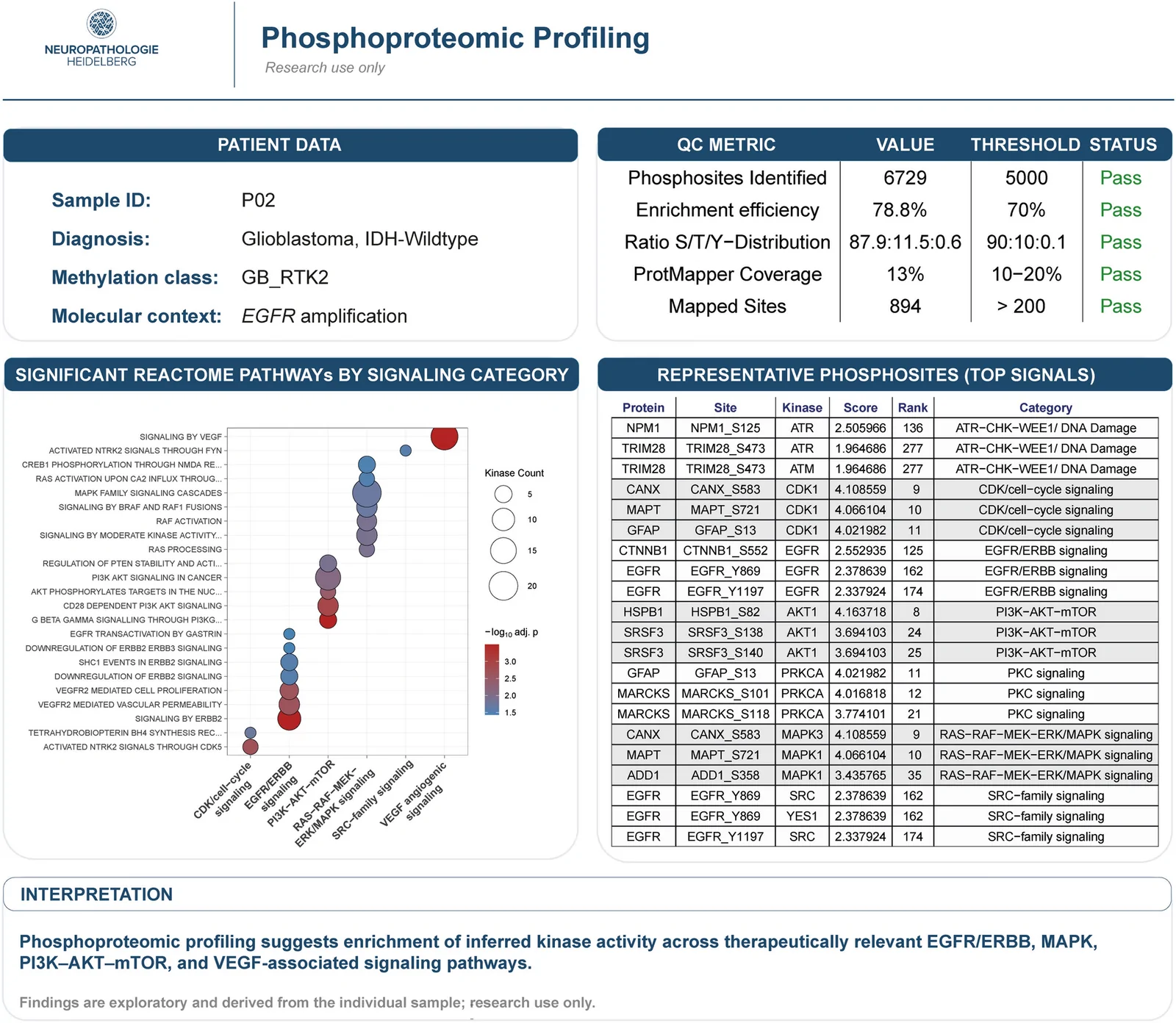
Prototype phosphoproteomic report integrating quality control, pathway enrichment, and representative phosphosite findings for an individual glioblastoma. The report illustrates a potential framework for case-level interpretation of phosphoproteomic data using an illustrative glioblastoma, IDH-wildtype (P02; DNA methylation class GB_RTK2; *EGFR* amplification), as an example. Quality control metrics summarize phosphosite identification depth, phosphopeptide enrichment efficiency, serine/threonine/tyrosine (S/T/Y) distribution, ProtMapper coverage, and the number of mapped sites. Significant Reactome pathways are grouped into predefined signaling categories; dot size represents the number of contributing kinases and color indicates the −log₁₀ adjusted P value. Representative pathway-associated phosphosites are shown together with the associated kinase, score, rank, and signaling category. The integrated interpretation suggests enrichment of inferred kinase activity across therapeutically relevant EGFR/ERBB, RAS–RAF–MEK–ERK/MAPK, PI3K–AKT–mTOR, and VEGF-associated signaling pathways. Findings are derived from the individual tumor without reference to the remaining cohort and are intended for research use only; they do not imply therapeutic sensitivity

To illustrate how phosphoproteomic information could be summarized at the level of an individual tumor, we generated a prototype research-use-only report for one representative *EGFR*-amplified glioblastoma (P02; DNA methylation class GB_RTK2; Fig. 4). Importantly, the report was generated from the phosphoproteomic profile of the individual tumor without requiring a reference cohort. The report combines analytical quality-control metrics with pathway-level enrichment and representative phosphosite findings (Supplementary Table 6). In this example, phosphoproteomic profiling suggested enrichment of inferred kinase activity within therapeutically relevant signaling pathways, including EGFR/ERBB, RAS–RAF–MEK–ERK/MAPK, PI3K–AKT–mTOR, and VEGF-associated signaling. Multiple pathway-associated phosphosites, including EGFR Y869 and Y1197, supported these pathway-level findings. The report is intended to illustrate a potential framework for integrating phosphoproteomic information into molecular reporting rather than to provide a validated diagnostic or therapeutic readout.

## Discussion

This proof-of-concept study demonstrates that proteomic and phosphoproteomic profiling of routine FFPE glioblastoma tissue can provide biologically coherent functional information that can be interpreted alongside established genomic and epigenomic diagnostics. Previous methodological studies have established that quantitative phosphoproteomic analysis is feasible in FFPE tissue, and recent developments have substantially increased analytical depth and throughput [7, 22]. Building on these advances, our study applies phosphoproteomic profiling to comprehensively characterized neuropathological specimens and uses a defined genomic alteration as a biological benchmark to assess how functional signaling information complements established molecular diagnostics. In this setting, routine FFPE material yielded robust phosphoproteomic profiles that could be directly related to genomic and epigenomic tumor characteristics.

A central observation was the recovery of biologically expected molecular changes associated with *EGFR* amplification across both protein abundance and phosphorylation levels. Global proteomic analysis demonstrated increased EGFR protein abundance in *EGFR*-amplified tumors and revealed broader group-associated pathway differences. At the phosphoproteomic level, protein-abundance-adjusted analysis identified multiple phosphorylation differences associated with *EGFR* amplification, including enrichment of EGFR Y1110 and Y1197, while kinase-substrate enrichment indicated coordinated EGFR- and SRC-family-associated signaling. These findings are consistent with previous proteogenomic studies showing that genomic alterations in glioblastoma are associated with downstream changes at the protein and phosphorylation levels. Large-scale proteogenomic profiling of treatment-naive glioblastomas has demonstrated that integration of genomic, proteomic, and post-translational modification data can reveal pathway-level consequences of genomic alterations that are not captured by genomic information alone [23]. Longitudinal proteogenomic analyses further demonstrate that glioblastoma signaling states can undergo substantial post-translational remodeling during tumor evolution, emphasizing that functional state cannot be inferred from genomic alterations alone [10]. Other integrated proteomic and phosphoproteomic analyses of IDH-wildtype glioblastoma have likewise demonstrated substantial molecular heterogeneity and identified signaling patterns with potential therapeutic relevance [13]. Our findings extend this relationship between genotype and signaling state to routinely processed FFPE material, using *EGFR* amplification as a defined genomic reference point.

Previous proteomic and phosphoproteomic studies have also demonstrated that glioblastomas can be stratified into functionally distinct molecular subgroups. Integrated proteomic and phosphoproteomic analyses have identified tumor states characterized by distinct signaling networks and kinase dependencies, including subtype-associated PKCδ and DNA-PK activity [12]. These findings support the concept that functional proteomic information may complement genomic classification by resolving biologically distinct signaling states. Whether such functionally defined signaling states provide prognostic information beyond established molecular classifiers will require evaluation in larger, clinically annotated cohorts. Importantly, *EGFR* amplification did not translate into a uniform phosphoproteomic phenotype. Protein-abundance-adjusted phosphoproteomic profiles showed only partial separation according to amplification status, consistent with substantial intertumoral heterogeneity. This observation illustrates the potential added value of phosphoproteomics for molecular characterization. Genomic alterations establish the presence of a molecular driver, whereas protein abundance and phosphorylation provide information on how that alteration is functionally expressed within an individual tumor. Tumors sharing the same genomic alteration may therefore differ substantially in downstream pathway utilization. Phosphoproteomic profiling may consequently provide an orthogonal functional layer that complements, rather than duplicates, genomic classification.

The translational relevance of this approach is supported by its compatibility with routinely archived FFPE material and its ability to interrogate signaling networks at the level of site-specific phosphorylation. Recent high-throughput workflows further demonstrate that substantial proteomic and phosphoproteomic depth can be achieved from clinically derived FFPE specimens [7]. Beyond technical feasibility, emerging precision-oncology frameworks increasingly explore how proteomic and functional signaling information can complement genomic findings in molecular tumor boards and therapeutic prioritization [8, 20]. The translational potential of phosphoproteomic profiling is further supported by studies in tumor entities beyond glioblastoma. In cholangiocarcinoma, phosphoproteomic profiling combined with computational analysis identified patient-specific signaling patterns and candidate drug sensitivities, with predicted responses subsequently supported in cellular models [9]. In hepatocellular carcinoma, phosphoproteomic profiling defined biologically and clinically distinct subtypes and identified candidate therapeutic vulnerabilities, including a kinase-targeted treatment that was subsequently evaluated in cell-line and patient-derived xenograft models [27]. Together, these studies illustrate how phosphoproteomic information may extend molecular characterization toward the functional prioritization of signaling pathways and therapeutic hypotheses. A clinically relevant example of functional pathway-based stratification is provided by the N^2^M^2^/NOA-20 umbrella trial, in which patients with glioblastoma and with activated mTOR signaling, as assessed by phospho-mTOR, were selected for treatment with temsirolimus [24]. This illustrates how assessment of pathway activation may provide therapeutic information beyond genomic alterations alone. More quantitative phosphoproteomic approaches could potentially extend such strategies by simultaneously assessing multiple signaling pathways, although prospective validation and clinically applicable thresholds will be required. At present, however, analytical complexity, infrastructure requirements, and costs of mass-spectrometry-based phosphoproteomics are likely to limit its application to selected clinical and translational settings. In addition, several challenges remain before phosphoproteomic information can be incorporated into routine neuropathological diagnostics. Kinase-substrate enrichment infers kinase activity from the coordinated behavior of annotated phosphosites rather than measuring enzymatic activity directly, and pathway-level associations require validation in larger independent cohorts and, where appropriate, by orthogonal approaches. In addition, standardization of tissue processing, analytical pipelines, quantitative thresholds, and reporting frameworks will be important for broader clinical implementation [1].

Several limitations of the present study should be considered. The cohort comprised only ten tumors and was intentionally centered on a single, well-characterized genomic alteration. Accordingly, individual differential phosphosites and inferred kinase activities should be regarded as hypothesis-generating rather than candidate biomarkers. Bulk tissue analysis also does not resolve spatial, cellular, or subclonal heterogeneity, which is particularly relevant in glioblastoma. Because phosphosite abundance is influenced by both total protein abundance and phosphorylation state, phosphosite intensities were adjusted for the abundance of the corresponding protein measured in the matched global proteome. This adjustment reduces the contribution of differences in total protein abundance to phosphosite-level changes; however, it does not constitute a direct measurement of phosphorylation stoichiometry. Finally, extension to larger cohorts and additional molecularly defined CNS tumor entities will be required to determine which functional signaling features are reproducible and of diagnostic or therapeutic relevance.

In conclusion, proteomic and phosphoproteomic profiling of routine FFPE glioblastoma tissue recovers biologically coherent molecular and signaling consequences associated with a defined genomic alteration while revealing functional heterogeneity beyond genomic classification. Rather than representing an alternative to sequencing, copy number analysis, or DNA methylation profiling, phosphoproteomics provides complementary information on the downstream functional state of the tumor. Integration of this functional layer with established molecular diagnostics may enable a more comprehensive characterization of CNS tumors that connects molecular identity with pathway activity.

## Supplementary Information

Below is the link to the electronic supplementary material.

Supplementary file1 (DOCX 255 KB)

Supplementary file2 (XLSX 10 KB)

Supplementary file3 (XLSX 15 KB)

Supplementary file4 (XLSX 795 KB)

Supplementary file5 (XLSX 460 KB)

Supplementary file6 (XLSX 83 KB)

Supplementary file7 (XLSX 24 KB)

## Data Availability

The processed proteomic and phosphoproteomic data generated in this study and the analysis code used for downstream data processing and statistical analyses are publicly available at https://github.com/FriedelDennisMNP/Functional_phosphoproteomic_profiling_of_routine_FFPE_CNS_tumor_tissue. Additional data supporting the findings of this study are provided in the Supplementary Tables.
