# Supplementary material for: Functional proteomic and phosphoproteomic profiling of routine FFPE CNS tumor tissue complements genomic and epigenomic characterization
Source: Acta Neuropathol. 2026 Sep 25;152(1):42. doi: 10.1007/s00401-026-03091-6 (PMC13615004; doi:10.1007/s00401-026-03091-6)
Supplement: Supplementary file 1 [file 401_2026_3091_MOESM1_ESM.docx]

**Functional proteomic and phosphoproteomic profiling of routine FFPE CNS tumor tissue complements genomic and epigenomic characterization**

**Supplementary Figure 1. Quality assessment of global proteomic profiling.** a) Number of confidently identified proteins per sample; the dashed line indicates the cohort median. b) Protein detection frequency across the cohort, showing the number of proteins detected in one to ten tumors. c) Distribution of log₂-transformed protein intensities across individual samples, showing comparable quantitative profiles across the cohort.
